# The Most Frequently Used Sequencing Technologies and Assembly Methods in Different Time Segments of the Bacterial Surveillance and RefSeq Genome Databases

**DOI:** 10.3389/fcimb.2020.527102

**Published:** 2020-10-19

**Authors:** Bo Segerman

**Affiliations:** ^1^Department of Microbiology, National Veterinary Institute (SVA), Uppsala, Sweden; ^2^Department of Medical Biochemistry and Microbiology, Uppsala University, Uppsala, Sweden

**Keywords:** sequencing technologies, bacterial genomes, surveillance, RefSeq, assembly methods

## Abstract

Whole genome sequencing has become a powerful tool in modern microbiology. Especially bacterial genomes are sequenced in high numbers. Whole genome sequencing is not only used in research projects, but also in surveillance projects and outbreak investigations. Many whole genome analysis workflows begins with the production of a genome assembly. To accomplish this, a number of different sequencing technologies and assembly methods are available. Here, a summarization is provided over the most frequently used sequence technology and genome assembly approaches reported for the bacterial RefSeq genomes and for the bacterial genomes submitted as belonging to a surveillance project. The data is presented both in total and broken up on a per year basis. Information associated with over 400,000 publically available genomes dated April 2020 and prior were used. The information summarized include (i) the most frequently used sequencing technologies, (ii) the most common combinations of sequencing technologies, (iii) the most reported sequencing depth, and (iv) the most frequently used assembly software solutions. In all, this mini review provides an overview of the currently most common workflows for producing bacterial whole genome sequence assemblies.

## Introduction

Setting up a capacity to perform bacterial whole genome sequencing (WGS) requires many technical considerations. This mini review focuses on two important aspects (i) sequencing technology usage and (ii) genome assembly software usage. It summarizes and briefly describes methods which are frequently reported as being used in the RefSeq and surveillance sections of the GenBank bacterial genome database. A historical perspective is also given by comparing different time segments of the databases. Thus, the focus of this mini review is to use the representation in the genome databases to give an overview of sequencing technologies and assembly methods that are or have been in active use specifically for bacterial WGS.

Genome sequences are typically determined by a shotgun approach where sequence reads are generated from random places in the genome using one out of a limited number of available sequencing technologies (Heather and Chain, 2016). The technologies are often divided into second generation sequencing that produce large amounts of short sequence reads (up to a few hundred nucleotides) and third generation sequencing that produce fewer but much longer sequence reads (tens of thousands of nucleotides) (Heather and Chain, 2016). The long sequence reads are also associated with less accuracy in calling the individual bases. To correct these errors, either a hybrid approach combining long read data with short read data or a self-correction using a consensus approach is usually applied (Fu et al., 2019).

Some types of WGS analysis are done directly on the sequence read data (e.g., calling single nucleotide polymorphisms, SNPs, by mapping the reads to a reference sequence), but often an assembly software is used to create a genome assembly to be used in downstream analysis (e.g., core genome multilocus sequence typing, cgMLST, and antimicrobial resistance, AMR, gene identification) (Schurch et al., 2018). The number of assembly software solutions available is much larger than the number of sequence technologies and may be hard to overview in an unbiased way. This mini review aims to give a wide-ranging summarization of assembly software solutions actively in use for bacterial genomes based on their reported usage in the genome databases.

The function of an assembly software is to attempt to create a representation of the actual genome from the raw sequencing read data which represent fragmented pieces of the genome with each genomic region on average covered multiple times (Simpson and Pop, 2015; Sohn and Nam, 2018). The resulting genome assembly consists of a variable number of continuous sequences referred to as “contigs” that together represents most of the genome. Some parts of the genome usually remains unresolved in the form of gaps between the contigs. Techniques such as paired end sequencing (generating paired sequence reads known to be in close proximity as they originate from opposite ends of the same short DNA fragment) or mate pair sequencing (paired sequence reads from opposite ends of longer fragments) can generate information that can link contig ends via a stretch of unknown sequence, a spanned gap. An assembly containing spanned gaps between at least some of the contigs are typically submitted as “scaffolds.” Some genome projects resolves all gaps and uncertainties resulting in a “complete genome.” A complete genome is gapless, have no runs of ten or more ambiguous bases and contains all expected chromosomes. Contigs, scaffolds and complete genomes represent different “assembly level” categories in the genome database. Some genome sequences are also submitted under the category “chromosome” which represent chromosome sequences that can have different levels of completion (https://www.ncbi.nlm.nih.gov/assembly/help/).

**Figure 1:**
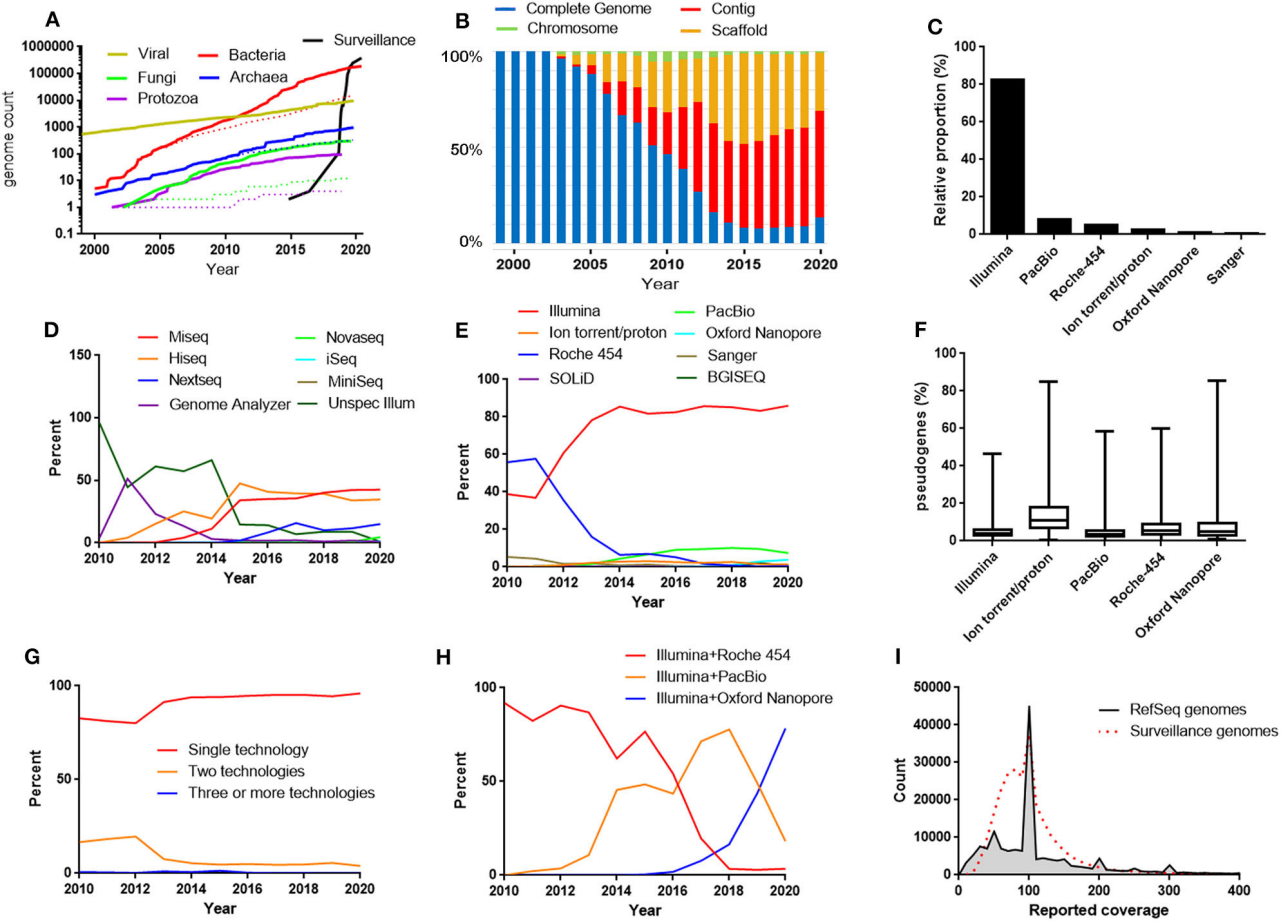
**(A)** The growth of the RefSeq microbial genomic databases and the database of bacterial genomes excluded from RefSeq for the reason “derived from surveillance project.” Dotted lines represent number of “complete genomes.” The data for 2020 includes genomes submitted before April 17. **(B)** Relative proportions between the different assembly levels in the bacterial RefSeq genome database. **(C)** The most frequently used sequencing techniques in the bacterial RefSeq database. **(D)** Relative proportions between the different Illumina platforms in the bacterial RefSeq genome database. **(E)** Relative proportions between sequencing techniques used in bacterial RefSeq divided by years. **(F)** Frequencies of pseudogenes in bacterial RefSeq genomes reported to be produced by one technique alone. **(G)** Relative proportions between genomes produced by a single sequencing technique and combinations of techniques. **(H)** Relative proportions between the most frequently used combinations of sequencing techniques in the bacterial RefSeq genome database. **(I)** Histogram of the reported sequence depth (coverage) used in the bacterial RefSeq genome database and in the bacterial surveillance project genome database.

The bacterial RefSeq genome database contains genomes from the bacterial GenBank genome database that remains after filtering out sequences that do not fulfill certain criteria, originally mostly related to quality or purity-concerns (https://www.ncbi.nlm.nih.gov/assembly/help/anomnotrefseq/). Genome sequences produced in surveillance projects are also not included among the RefSeq genomes and this part of the genome database is currently the one with the fastest growth and has in just a few years become larger than the bacterial RefSeq genome database (Figure 1A).

In the early days of genome sequencing, the proportion of completed genomes was high, but it rapidly started declining as a result of a fast growing contig and scaffold level genome sequence production (Figure 1B). However, in the latest years a trend has become visible that the contig/scaffold level is no longer continuing to outcompete the complete genome category (Figure 1B). This is perhaps related to increased usage of long read sequencing technologies that facilitates the gap closure procedure. Approximately 90% of the genomes completed during 2019 were labeled as having made use of long read sequencing, either PacBio or Oxford Nanopore.

The list of technologies and assembly software solutions and their relative usage presented in this mini review was derived by downloading and summarizing the information in the “^*^_assembly.stats.txt” files from over 430,000 bacterial genomes (RefSeq + surveillance genomes). The “^*^_feature_count.txt” files were also used from over 170,000 genomes (RefSeq). The data was downloaded from National Center for Biotechnology Information (NCBI)(ftp://ftp.ncbi.nlm.nih.gov/genomes) during Nov 2019 and represent the status of the database at the 6th of Nov 2019 (RefSeq) and 27th of Nov (surveillance genomes). During revision, an update of the analysis was made to also obtain data for the first 4.5 months of 2020. The reported sequencing technology and assembly method information was summarized and analyzed. Alternative naming and misspelling cases were merged by making lists of name-aliases for each technology/assembly method.

## Sequencing Technologies

Currently, 82% of the bacterial genomes in RefSeq were produced by the short read Illumina sequencing technology (Figure 1C). Among the surveillance genomes, the Illumina dominance was further more pronounced. The genomes were in 99.9% of the cases produced by Illumina technology. The technology was originally developed by a company called Solexa (Cronn et al., 2008), which was acquired by Illumina 2007 (Solexa was considered as an alias for Illumina in this analysis). Illumina sequences are produced by attaching adapters to the end of short DNA fragments followed by a bridge amplification step and finally the sequences are determined by sequencing by synthesis, one nucleotide at a time, with fluorescently tagged dNTPs (Heather and Chain, 2016). The accuracy of each base is high but the read length is a few hundred bases at the most. A number of different machines with different throughput are available. In the bacterial RefSeq database, the most frequently used Illumina machines for bacterial WGS were HiSeq and MiSeq (Figure 1D).

The second largest technology was the long-read technology PacBio, also known as Single Molecule Real-Time (SMRT) sequencing. Together PacBio and Illumina makes up over 90 % of the genome sequences in RefSeq. PacBio is a long read technology that is based on monitoring the activity of DNA polymerase molecules attached to the bottom surface of nano-sized sequencing units called zero-mode-waveguides (ZMWs) using fluorescent labeled nucleotides (Heather and Chain, 2016).

In the beginning of bacterial WGS, a technology based on emulsion PCR followed by pyrosequencing provided by 454 Life Sciences (Margulies et al., 2005), acquired by Roche 2007 made the largest contribution to RefSeq microbial genomes (Figure 1E). This was at least partly because this technology offered longer read lengths compared to the competitors making the assembly process more efficient. However, within a few years the popularity of Roche-454 started to decline likely because of its higher cost per sequenced base and because Illumina sequencing had improved their sequencing read length. In 2013 Roche announced the discontinuation of the Roche-454 sequencing platform.

The Ion-torrent/proton systems (today sold by Thermo Fisher) has similarities to 454 sequencing but uses microwells on a semiconductor chip to measure changes in pH during the sequencing cycles instead of pyrosequencing (Heather and Chain, 2016). Ion-torrent/proton steadily contributes to a small part (1–3%) of the genomes. In Roche-454 and Ion-torrent/proton, each sequencing cycle do not read one single base at a time but instead reads all constitutive bases of the same type. Because of this, the Roche-454 and the Ion-torrent/proton technologies are known to be prone in errors determining homopolymer lengths. This may lead to incorrect frameshifts when annotating the genomes resulting in false pseudogenes. To investigate if different sequencing techniques are associated with different pseudogene frequencies in the RefSeq database, the frequency of genes annotated as pseudogenes were plotted for the assemblies produced solely by one of the most common sequencing techniques (Figure 1F). This illustrates that especially the Ion torrent/proton assemblies has clearly elevated levels of pseudogenes compared to the others. Furthermore, examining the bacterial genome sequences excluded from RefSeq for the reason “many frameshifted proteins,” Ion torrent/proton was strongly overrepresented constituting over 50% of the cases.

The long read sequencing technology Oxford Nanopore (MinIon/GridION/Flongle) also makes out a small fraction but is gradually increasing and constituted 2019 around 3% of the sequences. In Oxford Nanopore sequencing electric signals are measures as the DNA is passed through a nanopore (Heather and Chain, 2016). Oxford Nanopore sequencing does not require an expensive machine such as for PacBio sequencing, but instead uses a small device that can be connected to a computer via a USB interface.

An unspecified form of BGISEQ also entered the list at around 2% 2019 by means of a batch submission from BGI. SOLiD sequencing (Sequencing by Oligonucleotide Ligation and Detection—today sold by Thermo Fisher) is present in the database but has never been a frequently used technique for producing bacterial whole genome assemblies, probably because the sequence length is too short for making efficient assemblies. Some older sequences are derived by solely (first generation) Sanger sequencing. Helicos single molecule sequencing has been used in a handful of genome assemblies. However, Helicos Biosciences filed for bankruptcy 2012. A few genome assemblies mention use of OpGene, which probably reflects usage of optical mapping to facilitate scaffolding of contigs.

To improve the assembly, a combination of sequencing techniques can be used. However, the vast majority of the sequences are reported to be produced with a single technique (Figure 1G). Traditionally, targeted Sanger sequences has been used to complement weak spots in the assembly. However, the most frequent combination of techniques in RefSeq is Illumina and PacBio. Illumina and Roche-454 has historically been a very popular combination and still ranks as second place. Looking on a per year basis shows that Illumina and Pacbio has gradually taken over from Illumina and Roche 454. However, a recent trend is that PacBio data is used alone and is less frequently combined with Illumina. Instead, Illumina and Oxford Nanopore has started to take over as the most common combination (Figure 1H).

The genome coverage that submitters reported for their assemblies were also summarized (Figure 1I). The coverage typically lies in the range 30-150X and peaks are visible at 50X and 100X. This may be due to down-sampling strategies aiming at these coverages in the assembly pipelines.

## Assembly Methods

**Figure 2:**
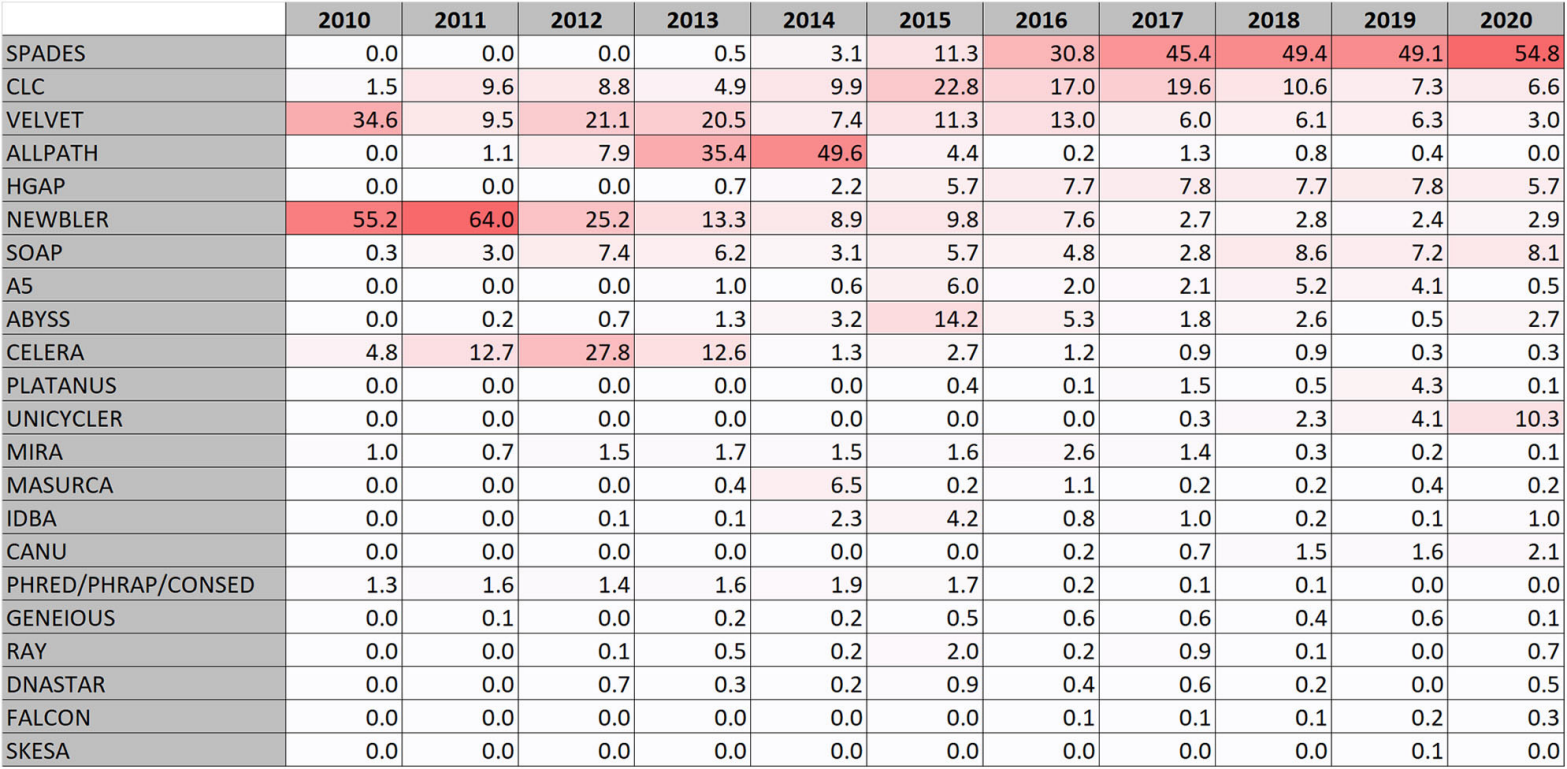
Heatmap of the most frequently used genome assembly software solutions used.

In the early days of the genome database development, Newbler (also known as GS *de novo* assembler) (Margulies et al., 2005) was the most used assembly software. It was designed for Roche-454 sequence data which was the most frequent form of data (Figure 1E). The software was developed by 454 Life sciences and later maintained by Roche. As Roche 454 sequencing fell in popularity, Newbler usage fell as well, but is still being used at a low level (Figure 2). Also the Celera assembler (Myers et al., 2000) developed by Celera for their drosophila and human genome projects has been used for bacterial genomes. Some reports using Phread/Phrap/Consed (Gordon et al., 1998) are present. MIRA is also a software that was used early for assembling bacterial genomes. It was originally developed in a PhD project (http://www.ub.uni-heidelberg.de/archiv/7871).

The early assembler programs typically analyze overlaps between whole sequence reads to build a consensus. The more recent assembler programs, at least for second generation reads, generally uses methods that divides the reads into *k*-mers and creates de Bruijn graphs (Pevzner et al., 2001). The first de Bruijn graphs based assembler appearing in the genome database was VELVET that was described 2008 (Zerbino and Birney, 2008) and it is still being used (Figure 2).

In the years 2013 and 2014 ALLPATHS/ALLPATHS-LG (Butler et al., 2008; Maccallum et al., 2009; Gnerre et al., 2011) became the most frequently used assembly software (Figure 2). This was mainly due to a large sequence submission activity of Broad Institute of mainly *Staphylococcus aureus* and *Mycobacterium tuberculosis*. The ALLPATHS developers subsequently switched to the DISCOVAR project but it never became frequently used for bacterial genomes.

However, in the latest years a clear trend has emerged that SPAdes (Bankevich et al., 2012) has raised up as the most frequently used assembly software and is in total the most used assembly software in RefSeq. The popularity of SPAdes cannot be explained by a few large sequence producers, it is used by many. It is at the time of writing this actively being maintained and new improved versions are frequently being released.

Looking at the surveillance genomes, ~98% of them were assembled by the program SKESA (Souvorov et al., 2018) developed by NCBI, followed by SPAdes (Bankevich et al., 2012) at only 1.5%. SKESA had at this point only a minor representation in RefSeq (Figure 2).

Some genomes are assembled by an assembly software in a commercial software suite. The largest actor in this segment of the database is CLC that include a de Bruijn graph assembler. CLC bio was acquired by QIAGEN in 2013. Other commercial software suits used for assembling bacterial genomes include DNASTAR lasergene, GENEIOUS, and EvoCAT from Evogene.

In addition, a number of mainly de Bruijn graph assemblers with low but relatively consistent usage exists (Figure 2). These include: (i) SOAPdenovo (Li et al., 2010; Luo et al., 2012) which is an assembler for both large and small genomes. There was a follow up project called MEGAHIT (Li et al., 2015), but it was more aimed for assembling metagenomes. (ii) ABYSS, Assembly By Short Sequences (Simpson et al., 2009) which uses a parallelized algorithm. (iii) A5/A5-miseq (Tritt et al., 2012; Coil et al., 2015) which is short for Andrew And Aron's Awesome Assembly pipeline that make use of the IDBA assembler. IDBA is also used alone and is short for Iterative de Bruijn graph assembler (RECOMB 2010, https://doi.org/10.1007/978-3-642-12683-3_28). (iv) MaSuRCA which stands for The Maryland Super Read Cabog Assembler (Zimin et al., 2013) and a hybrid form of this assembler (Zimin et al., 2017) uses an approach creating “super reads.” (v) PLATANUS (Kajitani et al., 2014, 2019) which stands for PLATform for Assembling NUcleotide Sequences, and finally (vi) RAY (Boisvert et al., 2010) designed for using mixed sequencing technologies.

When looking at long read data, PacBio reads are mostly assembled by HGAP (Chin et al., 2013) (73%, 2020) developed by Pacific Biosciences followed by Canu (Koren et al., 2017) at 13%. Canu is a fork of the Celera assembler. Oxford Nanopore data are mostly assembled with Unicycler (Wick et al., 2017) (47%, 2020) and Canu (Koren et al., 2017) (36%). Unicycler processes Illumina data using SPAdes and can also be run with Illumina data only. Less used assembly programs used with long read data include Flye (Kolmogorov et al., 2019), SPAdes, SOAP, Falcon (a diploid aware version of HGAP), and Hinge (Kamath et al., 2017) which is optimized for repeat resolution.

## Final Conclusions

The genome databases continue, year after year to grow vastly. It is becoming an extensive big data resource. The massive burst of the surveillance genomes is also worth noticing. This is an effect of the new trend that NGS is replacing traditional typing methods such as Sanger sequencing based multilocus sequence typing (MLST) and Pulsed-field gel electrophoresis (PFGE) (Nadon et al., 2017; Ribot et al., 2019) (https://www.ecdc.europa.eu/en/publications-data/ecdc-strategic-framework-integration-molecular-and-genomic-typing-european). The migration to whole genome sequencing has though only just begun and a large expansion is still expected. The workflow for handling surveillance related NGS data is still under formation and it is still too early to draw detailed conclusions about how this emerging data resource will be constituted. However, massive amounts of NGS data from bacterial genomes of the major human pathogens will most certainly be produced in the years to come.

In summary, the vast majority of the genome sequences are produced by Illumina sequencing at 30X-150X coverage. Long read sequencing is on the rise and probably contributes to more completed genomes being produced but can still not compete if the aim is to produce massive amount of low-cost genomes. Roche-454 sequencing was initially a major player but has effectively disappeared. The Ion-torrent/Ion-proton technology makes out a steady but low percentage but it is struggling with quality problems of the final assembly, especially concerning homopolymers. This technology therefore appears to be less competitive in analysis workflows that requires high quality whole genome assemblies to be produced (such as cgMLST). Nevertheless, the technique is fast and can be competitive when using other whole genome analysis approaches such as SNP analysis workflows, where low quality regions/indel errors can be filtered away without obstructing the analysis.

The most popular assembly program in RefSeq is today SPAdes (Bankevich et al., 2012). The surveillance genome submissions typically goes through the NCBI assembler SKESA (Souvorov et al., 2018). PacBio data are currently assembled mostly with HGAP and Oxford Nanopore data with Unicycler or CANU. Pre-assembly steps such as read trimming is very seldom reported but are probably often carried out. Likewise, post assembly processing steps aiming to correct technical errors/polish, scaffold, merge, combine or in other ways process already assembled contigs are only sporadically reported in the database.

Depending on base composition and the nature of the repetitive parts of the genome analyzed, the optimal analysis is probably somewhat different in different species. Benchmarking/proficiency tests are needed to find the optimal solutions for each laboratory. The aim of this mini review is to provide a broad listing, unbiased and free from personal opinions, of technologies and particularly assembly methods which are being actively used to assemble bacterial genomes. This can be used as basis for setting up comparisons of workflows specialized for the requirements in each individual lab.

## Author Contributions

BS conceptualized, prepared the data and wrote the manuscript.

## Conflict of Interest

The author declares that the research was conducted in the absence of any commercial or financial relationships that could be construed as a potential conflict of interest.
